# Environmental and occupational bronchiolitis obliterans: new reality

**DOI:** 10.1016/j.ebiom.2023.104760

**Published:** 2023-08-18

**Authors:** Sergey S. Gutor, Robert F. Miller, Timothy S. Blackwell, Vasiliy V. Polosukhin

**Affiliations:** a Division of Allergy, Pulmonary and Critical Care Medicine, Department of Medicine, Vanderbilt University Medical Center, Nashville, TN, USA; b Veterans Affairs Medical Center, Nashville, TN, USA

**Keywords:** Review, Bronchiolitis obliterans, Constrictive bronchiolitis, Vasculopathy, Inflammation, Fibrosis

## Abstract

Patients diagnosed with environmental/occupational bronchiolitis obliterans (BO) over the last 2 decades often present with an indolent evolution of respiratory symptoms without a history of high-level, acute exposure to airborne toxins. Exertional dyspnea is the most common symptom and standard clinical and radiographic evaluation can be non-diagnostic. Lung biopsies often reveal pathological abnormalities affecting all distal lung compartments. These modern cases of BO typically exhibit the constrictive bronchiolitis phenotype of small airway remodeling, along with lymphocytic inflammation. In addition, hypertensive-type remodeling of intrapulmonary vasculature, diffuse fibroelastosis of alveolar tissue, and fibrous thickening of visceral pleura are frequently present. The diagnosis of environmental/occupational BO should be considered in patients who present with subacute onset of exertional dyspnea and a history compatible with prolonged or recurrent exposure to environmental toxins. Important areas for future studies include development of less invasive diagnostic approaches and testing of novel agents for disease prevention and treatment.

**Outstanding questions**

• Environmental/occupational BO is a heterogeneous clinical syndrome that can result from chronic, recurrent exposure to a broad spectrum of inhaled toxins and irritants.
• Modern cases of environmental/occupational BO are often characterized by indolent evolution of respiratory symptoms without a history of high-level, acute exposure to airborne toxins.
• Exertional dyspnea is the most common respiratory symptom in patients who present with environmental/occupational BO.
• While some patients with environmental/occupational BO present with abnormalities on pulmonary function testing and/or chest CT scans, others present without diagnostic findings on standard, non-invasive clinical evaluation.
• Lung biopsies of patients with environmental/occupational BO typically exhibit pathological findings in all distal lung compartments, including small airways, pulmonary vasculature, alveolar tissue, and visceral pleura.

## Introduction

*Bronchiolitis Obliterans* (BO), also known as obliterative bronchiolitis (OB), was first identified in individuals acutely exposed to high levels of toxic gases, which resulted in the rapid onset of non-cardiogenic pulmonary edema, followed by progressive dyspnea and airflow obstruction in survivors.^1^ BO was then described in some patients with autoimmune disease^2^^,^^3^ and as a complication of solid organ transplantation (particularly lung)^4^ or hematopoietic stem cell transplantation.^5^

Over the past two decades, a number of new case series of BO have been reported in relationship to a variety of environmental/occupational exposures, including flavoring/popcorn factory workers,^6^, ^7^, ^8^ survivors and responders of the World Trade Center (WTC) collapse in 2001,^9^, ^10^, ^11^ and U.S. soldiers deployed to Southwest Asia and Afghanistan.^12^^,^^13^ An important distinction from historical BO cases is that these more recent cases were associated with subacute or chronic exposures to environmental/occupational toxins and manifested primarily as indolent onset of exertional dyspnea, cough, and exercise limitation. Many of these patients were difficult to diagnose because they presented with normal (or mildly abnormal) pulmonary function testing (PFT) and had minimal findings on high-resolution computed tomography (HRCT) of the chest.^14^ Histopathological analysis of lung biopsies from these patients showed multicompartmental pathology in the distal lung, including small airways fibrous remodeling, diffuse fibroelastosis in alveolar tissue, vasculopathy, and inflammation/fibrosis of the visceral pleura.^7^^,^^10^, ^11^, ^12^, ^13^^,^^15^ Together, these findings support the emerging concept of a milder, subacute form of occupational/environmental BO without clear diagnostic findings on non-invasive clinical evaluation. In this review, we will focus on updating our understanding of the etiology, clinical features, and pathobiology of environmental and occupational BO.

## Historical descriptions of bronchiolar disorders

The earliest cases of BO from environmental exposures were reported in the relation to acute exposures of healthy subjects to nitrogen dioxide as “bronchiolitis fibrosa obliterans” (BFO). This pathological entity was first described in 1901 and defined as obstruction of bronchioles by luminal accumulation of loosely-organized granulation tissue in the absence of other lung fibrosing conditions, such as interstitial pneumonias.^16^ This process was conceptualized as a three-stage process.^17^ The first stage, typically several days in duration, was defined by severe dyspnea, cyanosis and cough, but without fever. The second stage consisted of an asymptomatic latency period followed by the third stage where respiratory symptoms increased, along with progressive airflow obstruction and production of bloody sputum.^17^^,^^18^ Generally, symptoms related to BFO developed in survivors ∼20 days after exposure. In BFO, distal bronchioles were reported to be filled by cell and tissue debris, with invasion of nascent granulation tissue extruding from the ulcerated walls of injured bronchioles. These polypoid masses of granulation tissue sometimes extended proximally into larger bronchioles and distally into alveolar tissue.^16^, ^17^, ^18^

Subsequent nomenclature divided BO into two groups based on whether the small airway pathology was primarily luminal or mural. One group was defined as *proliferative bronchiolitis* (PrB), which was characterized by the formation of intraluminal inflammatory polypoid buds of granulation tissue. This form of BO was reported in response to a variety of exposures, including silo gas,^19^^,^^20^ sulfur dioxide, hydrogen sulfide, and sulfur mustard gases used during World War I and the Iran–Iraq War in 1980–1988,^21^^,^^22^ zinc stearate,^23^ talcum powder,^24^ or as a consequence of respiratory viral infections.^25^^,^^26^ For many years, PrB was considered as either a pure small airway disease (without parenchymal involvement) or occurred in association with organizing pneumonia (OP), together known as BOOP.^27^ PrB is now most commonly categorized under the umbrella of OP or cryptogenic OP (COP) because it was realized that fibrotic material within the airway lumen most often extends from the alveolar compartment.^28^^,^^29^

Another type of BO was defined as *constrictive bronchiolitis* (ConB), which involves concentric fibrotic remodeling of the subepithelium that surrounds the airway lumen, resulting in extrinsic compression of affected bronchioles with luminal narrowing, mural infiltrate of mononuclear cells, and subepithelial granulation tissue.^30^ This type of BO was first described by Gosink et al.^31^ in 1973 and now predominates among environmental/occupational BO.

## Clinical characteristics of modern BO

### Environmental/occupational exposures

In contrast to the high-dose, acute exposures reported in most historical cases, BO in the current era usually results from sub-acute and sometimes mixed chronic exposure to environmental toxins. Cohorts of environmental/occupational BO have been reported in association with: 1) manufacturing, including diacetyl and other flavoring products associated with microwave popcorn production, flavoring manufacturing, coffee roasting and packaging^6^, ^7^, ^8^; 2) construction dust and debris in survivors and responders of the WTC collapse in 2001^9^, ^10^, ^11^; and 3) desert dust, burn pits and other environmental hazards in U.S. soldiers deployed to Southwest Asia and Afghanistan.^12^ In addition, small numbers of patients with BO have been reported in association with exposure to thionyl chloride used in battery production,^32^ fly ash (a powdery material composed mostly of silica generated from the burning of finely ground coal in a broiler),^33^ styrene in fiberglass-reinforced plastics workers,^34^^,^^35^ and powder material used in copy-machines.^36^ In rare cases, BO may develop without identifiable exposures and is designated as cryptogenic or sporadic BO.^37^

### Clinical presentation

Modern environmental/occupational BO features a spectrum of clinical presentations ranging from insidious onset of mild-moderate symptoms undetectable by non-invasive diagnostic tools to severe symptoms with obstructive/restrictive spirometry patterns and abnormal HRCT^14^^,^^38^^,^^39^; however, the major disease burden results from the mild-moderate form with indolent evolution of exertional dyspnea as the most frequent respiratory symptom. The lack of physiologic and imaging abnormalities using standard diagnostic approaches to evaluate dyspnea in these mild/moderate forms of BO make this disease perhaps one of the difficult diagnoses in respiratory medicine.^14^^,^^39^

Although the first cases of indolent, occupational-related BO were reported in patients with “silo-fillers lung”,^40^^,^^41^ the concept that BO could result from chronic, low-level exposure to environmental hazards was initially articulated in reports of cohorts of workers with diacetyl-related BO (also known as “popcorn lung” disease).^14^ Subsequently, biopsy-confirmed case series of BO with indolent ConB pathology were reported from WTC responders and survivors, U.S. soldiers returning from deployment to Iraq or Afghanistan, and Iranian survivors of sulfur mustard gas exposure.^10^, ^11^, ^12^, ^13^^,^^42^^,^^43^ These observations were summarized by Dr. Kathleen Kreiss, who first proposed that environmental/occupational BO can develop indolently and that the prevalence of exertional dyspnea is more common than spirometric abnormalities in exposed individuals.^14^

Clinical assessment of exposed individuals at one microwave popcorn plant showed increased prevalence of chronic cough, exertional dyspnea, and wheezing compared to expected rates after adjustment for age and smoking status.^6^ Of 116 symptomatic employees at one plant who underwent spirometric testing, most were in the normal range; however, 31 showed restriction, obstruction, or a mixed phenotype. Relatively similar data were obtained in investigations of other manufacturing facilities.^44^^,^^45^ While some of these individuals with significant obstruction on PFTs showed bronchial wall thickening and mosaic attenuation with air trapping on HRCT scans performed during expiration, most symptomatic popcorn workers had normal HRCTs, as well as normal PFTs.^14^

Clinical and epidemiologic assessment of affected lower Manhattan residents and WTC rescue and clean-up workers documented a variety of respiratory symptoms, including persistent cough, wheezing, shortness of breath, and exertional dyspnea.^46^^,^^47^ Of 1898 residents and workers who reported symptoms related to exposure to the dust, gas, and fumes released with the destruction of the WTC on September 11, 2001, dyspnea on exertion was reported in 67%, chronic cough in 46%, nasal or sinus congestion in 39%, chest tightness in 28%, and wheeze in 27% of participants. Abnormal spirometry was detected in 319 (29%), including obstruction in 67 (6%), restriction in 224 (20%) and a mixed phenotype in 28 (3%) cases. Notably, 90% of chest radiographs in 1679 study participants were reported as normal.^48^ Among 16 WTC responders/survivors with biopsy-confirmed bronchiolar pathology, seven patients showed “interstitial” findings with mild/moderate reticulation on HRCT, seven showed mosaic attenuation and air trapping, three showed ground glass opacities, and five patients were reported to have normal HRCT scans.^9^, ^10^, ^11^

The STAMPEDE III study (Study of Active Duty Military for Pulmonary Disease Related to Environmental Deployment Exposures) evaluated 380 Military Service members after deployment to Southwest Asia who reported chronic respiratory symptoms. This study revealed evidence of a variety of definable pulmonary conditions, including asthma in 87 patients (22·9%), laryngeal disorders in 57 patients (15·0%), excessive dynamic airway collapse in 16 patients (4·2%), interstitial lung disease in 6 patients (1·6%), fixed obstructive lung disorders in 11 patients (2·9%), isolated pulmonary function abnormalities in 40 patients (10·5%), and miscellaneous disorders in 16 patients (4·2%). The remaining 122 patients (32·1%) were classified as undiagnosed exertional dyspnea.^49^ In 2011, King and colleagues^12^ showed that lung pathology in soldiers with undiagnosed exertional dyspnea is frequently associated with the ConB phenotype on lung biopsy, despite the absence of clear abnormalities on HRCT scans and PFTs. Of the 38 soldiers with ConB in this study, 32 had normal spirometry and 25 had normal or minimally abnormal chest HRCT scans.^12^ While it is difficult to know for certain, we believe that it is likely that some undiagnosed cases in the STAMPEDE III study could have ConB.

In addition to patients with BO related to environmental or occupational exposures, some patients with sporadic BO have normal spirometry and HRCT chest scans. In one report of 19 patients with a pathological diagnosis of small airways pathology consistent with BO and unknown disease etiology, four had normal spirometry, 11 had obstruction, one had restriction, and one had a mixed pattern on PFT.^43^

Together, current data indicate that a substantial portion of patients with biopsy-proven ConB present with respiratory symptoms, particularly exertional dyspnea, in the absence of diagnostic findings on standard non-invasive clinical evaluation.

### Long-term outcomes

While some popcorn/flavoring workers showed progressive lung function decline after diagnosis, others have demonstrated disease stabilization within a few years after exposure cessation.^45^^,^^50^ Available follow-up data from 29 U.S. Veterans with biopsy-proven ConB (median follow-up period of 5 years) showed continued dyspnea on exertion in all 29 Veterans and persistent cough and/or chest tightness in 15 Veterans.^13^ While PFT parameters were in the normal range in most of these Veterans with ConB at follow-up, total lung capacity (% predicted) was reduced compared to studies done at the time of initial evaluation.^13^ Analysis of available biopsy samples in Iranians exposed to sulfur mustard gas showed ConB pathology more than two decades after exposure.^42^ Together, these limited data suggest long-term persistence of ConB pathology and respiratory symptoms in different cohorts of patients with variable environmental/occupational BO, even after cessation of toxic exposures.

## Histopathology of small airways in ConB

**Fig. 1:**
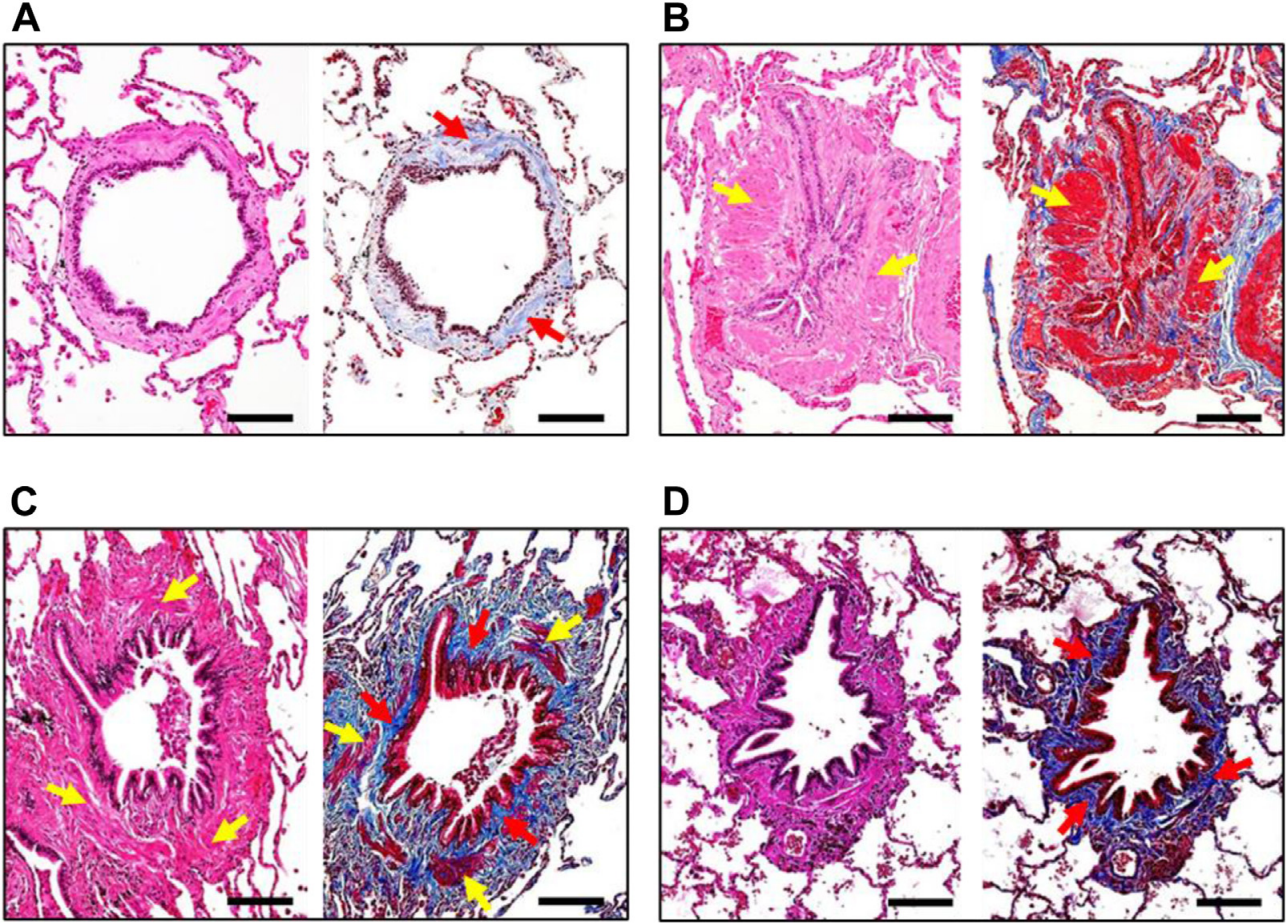
*Histopathological phenotypes of ConB in individual bronchioles*. A—normal-appearing bronchiole from non-diseased (ND) control subject. B—muscular phenotype; significant enlargement of smooth muscles (yellow arrows) in patient with sporadic ConB. C—mixed phenotype; subepithelial fibrosis and moderate enlargement of smooth muscles (yellow arrows) in U.S soldier with ConB. D—fibrosing phenotype; prominent subepithelial fibrosis without increased wall thickness in U.S soldier with ConB. Left images—hematoxylin and eosin stains; right images—Mason trichrome stains. Scale bars = 100 μm. These images were taken by Dr. Polosukhin and all authors confirm their originality.

As discussed above, ConB is the dominant small airways pathology in modern BO related to environmental/occupational exposures.^1^^,^^51^ Pathological findings of ConB include luminal narrowing in association with extrinsic compression by extensive subepithelial fibrosis, increased thickness of the lamina propria and/or smooth muscle hypertrophy. Based on histopathological manifestations, three distinct patterns with muscular, mixed and fibrosing phenotypes (Fig. 1) were described.^12^ Since all three phenotypes can be observed in the same case, they may correspond to different stages of the fibrosing process in ConB, rather than distinct pathological variants.

ConB can be identified on lung biopsy as a distinct pathology or combined with other small airways and lung parenchymal pathologies.^28^^,^^51^, ^52^, ^53^ The most frequent findings associated with ConB are cellular bronchiolitis, respiratory bronchiolitis, and bronchiolar metaplasia.

Pathological abnormalities in ConB are clearly separable from small airways disease associated with chronic obstructive pulmonary disease (COPD).^54^ While subepithelial fibrotic remodeling is present in both conditions, bronchiolar lesions in COPD are characterized by complex pathological abnormalities including frequent mucus luminal plugging, goblet cell metaplasia, neutrophilic infiltration, and progressive loss of supporting alveolar attachments, none of which is observed in ConB.^55^

## Modern ConB is a disorder that affects all distal lung compartments

**Fig. 2:**
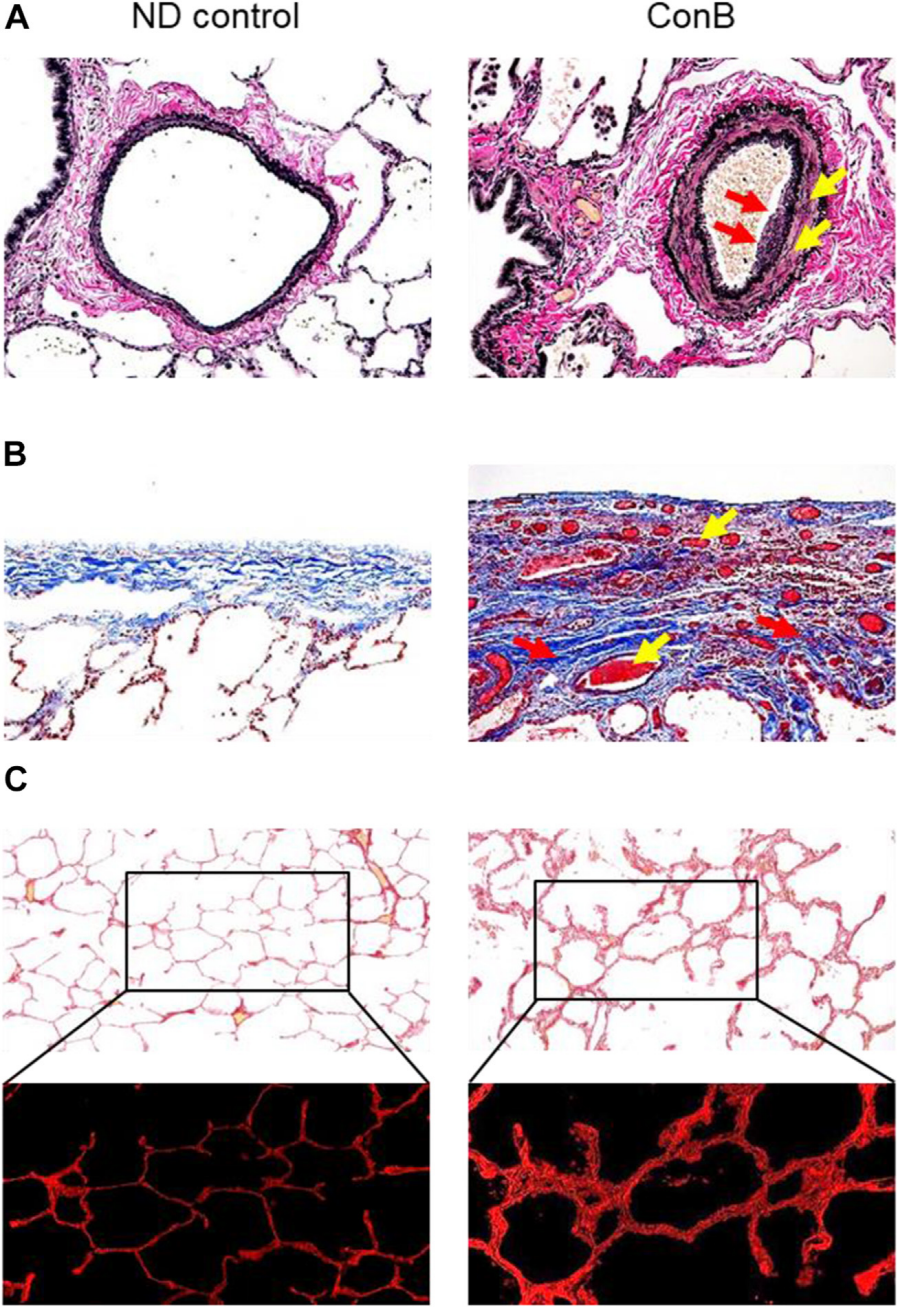
*Pathological abnormalities in pulmonary arteries and visceral pleura.* A—bronchovascular bundles; normal-appearing intrapulmonary artery from non-diseased (ND) control subject; moderate intima thickness (red arrows) and significant media thickness (yellow arrows) in pulmonary arteries in patient with sporadic ConB. Verhoeff-Van Gieson stain. B—visceral pleura; normal-appearing visceral pleura from ND control subject; inflammation, increased vascularity (yellow arrows), fibrosis (red arrows) and thickening of visceral pleura in patient with sporadic ConB. Mason trichrome stain. C—normal-appearing alveolar tissue from ND control subject; increased collagen content within inter-alveolar septa in patient with sporadic ConB. PicroSirius red stain (inserts demonstrate collagen fluorescence). Scale bars = 100 μm. These images were taken by Dr. Polosukhin and all authors confirm their originality.

Although ConB is classically defined as a pure bronchiolar fibrotic disorder,^1^^,^^30^ recent studies have suggested that the lung pathology in modern ConB is much broader than suggested by the name and is not limited by small airways but also extends on surrounding alveolar tissue and involves intrapulmonary vasculature and visceral pleura. Our group recently reported a comprehensive pathological analysis of lung biopsy tissue from 50 U.S. soldiers diagnosed with ConB after deployment to Iraq or Afghanistan and 8 patients with sporadic ConB.^13^^,^^54^ We found complex lung pathology involving all distal lung compartments (Fig. 2). In addition to ConB pathology, we identified: 1) diffuse lymphocytic inflammation; 2) diffuse interstitial fibrosis with fibroelastosis involving the inter-alveolar septa; 3) hypertensive-type intrapulmonary vascular remodeling; and 4) fibrosis, increased vascularization and thickening of visceral pleura. These findings were most striking in and around bronchovascular bundles, further implicating a pivotal role of bronchiolar injury in driving this disorder.^13^ Consistent with these findings, lung biopsies from individuals who developed chronic respiratory symptoms after exposure to WTC dust and fumes after 9/11 showed broad lung pathology with diffuse low-grade lymphocytic inflammation, small airway fibrous remodeling, vasculopathy and areas of interstitial fibrosis.^10^^,^^11^ Similarly, extensive lung pathology has been reported in other biopsy-proven ConB cohorts, including flavoring/popcorn factory workers,^7^^,^^8^ Iranian survivors of sulfur mustard gas exposure,^22^^,^^42^ and fiberglass workers.^35^ Importantly, findings outside the bronchioles are generally sub-radiographic.

Together, these data suggest that indolent ConB is often accompanied by extensive distal lung pathology affecting surrounding alveolar tissue, intrapulmonary vasculature, and visceral pleura. Based on this evidence, ConB might be considered as a bronchiolocentric distal lung disease in which small airways pathology is the hallmark and likely the initiating site of pathology.

## Pathogenesis of occupational and environmental ConB

ConB appears to be a common pathway of bronchiolar response to injury from a variety of insults.^1^ Growing evidence implicates airway epithelial injury and chronic intrapulmonary inflammation as central to disease pathogenesis.^1^^,^^30^^,^^54^ Cytokines, growth factors, and enzymes released by activated immune cells can promote fibroblast activation and excessive production and deposition of collagenous and non-collagenous extracellular matrix resulting in small airways fibrosis.^56^^,^^57^ Although intrapulmonary inflammation is likely a central driver of bronchiolar fibrosing pathology in environmental/occupational ConB, the mechanisms responsible for persistent intrapulmonary inflammation are largely unknown.

Immediate effects of inhaled particles and toxic gases on airway epithelial cells have been linked to oxidative stress through production of reactive oxygen species (ROS), which cause damage or modify cellular biomolecules, resulting in inflammation.^58^^,^^59^ Many toxic gases, including nitrogen dioxide or sulfur dioxide, can induce damage to airway epithelial cells via direct lipid peroxidation and induction of oxidative stress in affected cells.^60^, ^61^, ^62^ Exposure of differentiated human primary bronchial epithelial cells growing in air-liquid interface cultures to diacetyl vapor,^63^ aerosolized carbon nanoparticles^64^ or diesel exhaust particles^65^ results in significantly increased oxidative stress and expression of pro-inflammatory and tissue injury/repair markers.

Infiltration of airway walls, surrounding alveolar tissue, and pleura by T and B lymphocytes and formation of B cell-containing lymphoid follicles with or without germinal centers have been reported in ConB, including environmental and occupational variants.^13^^,^^43^^,^^54^ A detailed analysis of intrapulmonary inflammation in patients with sporadic ConB and in U.S. soldiers with ConB showed a striking Th1 pattern of lymphocyte gene expression.^54^ An important role for Th1 cytokines in ConB pathology has been reported in experimental studies.^66^^,^^67^ Together, available data indicate that chronic lymphocytic inflammation is a major feature of modern environmental/occupational ConB and may be essential for driving this pathology.

Fibrosis is a common outcome of many chronic inflammatory diseases. Although collagen production is a reversible part of wound healing, normal tissue repair can evolve into a progressively irreversible fibrotic response if the tissue injury is severe or repetitive, or if the wound-healing response itself becomes dysregulated.^56^ Rats exposed to diacetyl and 2,3-pentanideone showed damage to the basement membrane in small airways that predicted the degree of airway fibrosis.^68^^,^^69^ Based on these studies, we speculate that, depending on degree and extent of epithelial and basement membrane injury, resultant bronchiolar fibrosis may manifest as either intraluminal granulation tissue with loose connective matrix organization (as in wound healing) or subepithelial scarring with dense connective matrix organization. The first variant is associated with acute, high-dose exposures with severe epithelial and basement membrane injury, and formation of polypoid buds of granulation tissue (PrB pathology). In contrast, long-term, sub-acute inhalation exposures may induce milder, repetitive epithelial cell injury without severe basement membrane damage or destruction, resulting in persistent lymphocytic inflammation and progressive concentric subepithelial fibrosis (ConB pathology).

## Pathological determinants of dyspnea and exercise intolerance in BO

The correlation between pathology and physiology in patients with severe BO who present with significant obstruction or a combination of obstruction/restriction on PFTs appears straightforward. Given the pathological findings in lungs of patients with BO, it is likely that obstruction is related to luminal narrowing of small airways, whereas restriction is largely associated with fibrotic thickening of alveolar septa and visceral pleura, both of which could result in exertional dyspnea. In contrast, the pathological features accounting for symptoms in patients with mild/moderate BO in the absence of PFT abnormalities are less obvious and appear to be largely sub-radiographic.

One pathological feature related to ConB that has been largely overlooked until recently is vasculopathy. In this regard, we performed comprehensive histopathological evaluation of intrapulmonary blood vessels in 52 U.S. Veterans with biopsy-proven ConB and found prominent vascular remodeling at multiple anatomic levels including arteries from bronchovascular bundles, and intra-acinar arteries and veins.^70^ To determine whether vasculopathy in these Veterans could affect hemodynamics and underlie respiratory symptoms in these patients, we performed clinical evaluation of five Veterans with persistent dyspnea on exertion from this cohort and found that four out of five met criteria of pulmonary hypertension (PH) by right heart catheterization (RHC) according to the 2022 European Society of Cardiology/European Respiratory Society (ESC/ERS) guidelines.^71^ On transthoracic echocardiography, we identified borderline or overt right ventricle (RV) enlargement in three of these Veterans and reduced RV outflow tract (RVOT) acceleration time, a well-established surrogate measure of pulmonary pressure,^72^ in three out of five Veterans, while ventricular size and function were normal. Although further studies are needed, these data suggest that pulmonary vascular disease (PVD) could contribute to exercise related symptoms in BO patients.

Since complex lung pathology involving all distal lung compartments has been reported in several cohorts with occupational/environmental BO, it is tempting to speculate that a combination of pathological features contributes to dyspnea and exercise limitation in patients with mild/moderate BO. Since these features appear to vary in relative severity from patient to patient, it could be that varying degrees of airflow obstruction, reduced compliance, and vasculopathy underlie the persistent respiratory symptoms in patients with mild/moderate BO.

## Future directions

Standard diagnostic approaches for evaluation of dyspnea and exercise intolerance, including HRCTs, PFTs, echocardiograms, and standard cardiopulmonary exercising testing (CPET), have proven insufficiently sensitive to diagnose many cases of environmental/occupational BO. Although severe cases can be diagnosed based on an appropriate clinical setting of workplace or environmental exposures and supportive HRCT and PFT findings, these tests are mainly valuable to rule out alternative diagnoses in patients with mild/moderate BO who do not present with diagnostic findings on HRCT or PFTs. An important goal for the future is developing additional approaches with sufficient sensitivity to avoid the necessity of lung biopsy for diagnosis of ConB. As previously discussed, CPET combined with RHC holds promise to identify BO patients with mild PH at rest or exercise; however, the extent to which this approach is useful requires additional study.

Several newer technologies have shown promise for non-invasive diagnosis of BO. For example, measurement of respiratory impedance via a forced oscillation technique may be more sensitive than standard diagnostic tools for identifying small airways disease^73^ but have not been studied in patients with BO related to inhalational exposure. In addition, emerging radiographic technologies may assist in the diagnosis of BO. Parametric response mapping (PRM) is a computational CT analytic technique that measures differences in lung density using paired inspiratory and expiratory HRCT. A recent study found that military personnel with biopsy proven ConB have PRM analyses of HRCTs demonstrating increased small airways disease relative to healthy subjects.^74^ X-ray velocimetry is another radiographic modality that uses multi-dimensional fluoroscopic imaging with unique processing to quantitate regional lung tissue motion and lung ventilation. While this modality has received FDA clearance for use in COPD, CF and asthma, ongoing studies are evaluating its effectiveness in primary small airways disease.^75^

## Conclusions

During the last two decades, it has become clear that subacute environmental exposure to airborne toxins can cause a form of BO with ConB-type small airway remodeling, along with diffuse lymphocytic inflammation and a broad range of pathological abnormalities involving all distal lung compartments. Many of these cases present with an indolent evolution of exertional dyspnea and are often difficult to diagnose by routine, non-invasive clinical evaluation, making this diagnosis a significant challenge for Health Care providers. While the pathological basis for exertional dyspnea and exercise limitation in modern cases of BO is not always apparent, emerging data suggest that PVD may play an important role. As a result, hemodynamic measurements during exercise may prove to be a valuable diagnostic approach. Future investigations are needed to: 1) compare pathologic features of lung biopsies across a variety of toxic exposures, 2) determine the natural history of mild/moderate BO, 3) test new diagnostic modalities that can be broadly applied in suspected cases, and 4) generate new animal models to better understand disease mechanisms and test novel therapies for this under-recognized disorder.

**Search strategy and selection criteria**

We searched PubMed for articles published in from January 3, 2000, to January 20, 2023, with the search terms “bronchiolitis obliterans”, “constrictive bronchiolitis”, “small airways disease”, “inhalation exposures” and combinations of these terms with “pathogenic mechanisms” and “pathogenesis”. We selected the final list of references on the basis of relevance to the focus of this Personal View. Articles published in English, French, and German were included.

## Contributors

All authors contributed equally to the literature search and the writing and revision of this paper. All authors read and approved the final version of the manuscript.

## Declaration of interests

We declare no competing interests.
